# Statistical Evaluations of the Reproducibility and Reliability of 3-Tesla High Resolution Magnetization Transfer Brain Images: A Pilot Study on Healthy Subjects

**DOI:** 10.1155/2010/618747

**Published:** 2010-02-09

**Authors:** Kelly H. Zou, Hongyan Du, Shawn Sidharthan, Lisa M. DeTora, Yunmei Chen, Ann B. Ragin, Robert R. Edelman, Ying Wu

**Affiliations:** ^1^Pfizer Inc., New York, NY, USA; ^2^NorthShore University HealthSystem, Evanston, IL, USA; ^3^Albany Medical College, Albany, NY, USA; ^4^University of Florida, Florida, FL, USA; ^5^Northwestern University, Chicago, IL, USA; ^6^University of Chicago, Chicago, IL, USA; ^7^Center for Advanced Imaging, Department of Radiology, NorthShore University HealthSystem, 2650 Ridge Avenue, Walgreen G555, Evanston, IL 60201, USA

## Abstract

Magnetization transfer imaging (MT) may have considerable promise for early detection and monitoring of subtle brain changes before they are apparent on conventional magnetic resonance images. At 3 Tesla (T), MT affords higher resolution and increased tissue contrast associated with macromolecules. The reliability and reproducibility of a new high-resolution MT strategy were assessed in brain images acquired from 9 healthy subjects. Repeated measures were taken for 12 brain regions of interest (ROIs): genu, splenium, and the left and right hemispheres of the hippocampus, caudate, putamen, thalamus, and cerebral white matter. Spearman's correlation coefficient, coefficient of variation, and intraclass correlation coefficient (ICC) were computed. Multivariate mixed-effects regression models were used to fit the mean ROI values and to test the significance of the effects due to region, subject, observer, time, and manual repetition. A sensitivity analysis of various model specifications and the corresponding ICCs was conducted. Our statistical methods may be generalized to many similar evaluative studies of the reliability and reproducibility of various imaging modalities.

## 1. Introduction

Magnetization transfer (MT) imaging is a quantitative approach for detecting subtle or occult abnormalities in brain tissue. In previous studies, the Magnetization Transfer Ratio (MTR), an index of MT imaging, was sensitive to brain changes in patients with mild cognitive impairment, an Alzheimer's disease prodrome [1, 2], to new lesions in patients with multiple sclerosis, [3] and to changes associated with progression in chronic neurological disorders [4]. The higher magnetic field strength afforded by 3T allows MT image resolution to be augmented compared with conventional MT acquisition at 1.5T [5–7]. We developed a high resolution MT technique to detect subtle changes in anatomically small, functionally eloquent brain structures. The increased field strength affords whole-brain coverage with considerably thinner slices, potentially reducing partial volume artifacts. However, even among healthy subjects, numerous factors may introduce variability in measures derived from magnetic resonance (MR) data, such as static field *B*_0_ signal dropout and RF nonuniformity. Measurement variation may be introduced by scan repetitions, repositioning at different time points, and image post-processing. Moreover, 3T may be susceptible to variation associated with increased field strength [8]. Such variability may pose limitations when conducting clinical comparisons to differentiate normal and diseased brains or in developing statistically predictive algorithms. 

To validate high resolution MT for detecting early disease or for monitoring progression in chronic neurological disease, it is necessary to collect information on normative values and to evaluate the reliability and reproducibility of the measurements when measured across time in healthy controls. This investigation evaluated observer-agreement of high-resolution MT measurements determined from repeated brain scans of 9 healthy volunteers. We postulated that MT values would remain stable during the one month study interval. We evaluated the reliability and reproducibility of the high resolution MT measurements in 12 brain regions of interest (ROIs), applied statistical measures to the data and used complex multivariate mixed-effects models to test the statistical significance of several effects due to region, subject, observer, time, and manual repetition.

## 2. Materials and Methods

### 2.1. Study Subjects

The study was approved by the IRB at the North Shore University Health System, and conducted following the ethical principles outlined in the Declaration of Helsinki. Eleven healthy adult volunteers were randomly selected from a database maintained at the Center for Advanced Imaging, Radiology Department, NorthShore University Health System provided written informed consent and evaluated for eligibility criteria. To protect the subjects' confidentiality, all data were de-identified and handled according to the guidelines specified by the Health Insurance Portability and Accountability Act (HIPAA) in the USA.

### 2.2. Image Acquisition

Brain images were acquired using a 3T General Electric (GE) HDx system (Waukesha, WI, USA). Each volunteer was scanned twice in a randomly-selected time interval between 1 to 4 weeks. Methods for reducing random errors in image acquisition included the use of a body-coil for excitation to control B1 non-uniformities and an 8-channel quadrature receive-only coil [9]. MT pulses with (*M*_*s*_) and without saturation (*M*_0_) were applied at an offset frequency from water resonance. To accelerate the scan for whole-brain coverage, while maintaining thin slices, the image protocol was optimized based on 3T using 3D SPGR [5]. The Gaussian Sinc MT pulse was applied in 8 ms at a 1200 HZ offset. The stability of the scanner and set-up procedure were addressed with a fixed set of parameters per subject. MT pulse was based on a three-dimensional spoiled gradient recalled (3D SPGR) acquisition. The image protocol included the following parameters: TR 34 to 35 ms, TE 4 to 8 ms, imaging FA 5°, bandwidth 15.6 kHz, 0.75 NEX, phase FOV 0.75, voxel dimensions 0.9 × 0.9 × 0.9 ~ 1.3 mm^3^. The whole brain was covered in 90 to 140 slices with acquisition time ranging from 7 minutes 40 seconds to 10 minutes 20 seconds using a partial *k*-space acquisition.

### 2.3. Image Analysis

**Figure 1:**
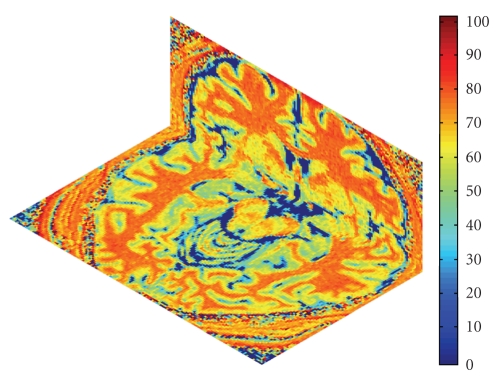
High resolution three-dimensional MTR map displayed both for the original view of the Axial plane and the reconstructed view of the Coronal plan. The MTR maps have excellent tissue conspicuity and high image resolution in all three dimensions.

**Figure 2:**
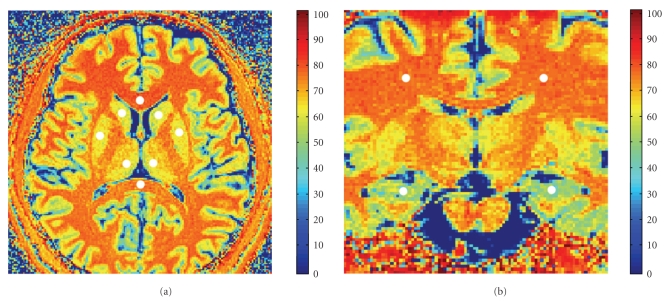
The Axial (a) and Coronal (b) views of high resolution MTR maps. Twelve brain ROIs are illustrated (white dots).

MTR maps were generated off-line on a General Electric AW Workstation (General Electric, Milwaukee, WI, USA) using the standard equation:

(1) $$\text{MTR}=\frac{M_{0}-M_{S}}{M_{0}}\times 100\text{\%},$$

where *M*_*S*_ and *M*_0_ were the signal intensities in a given voxel obtained, with and without the MT saturation pulse, respectively. MTR maps generated based on the high resolution MT are demonstrated in Figure 1. The 12 ROIs were: genu, splenium, left and right hemispheres of the hippocampus, caudate, putamen, thalamus, and cerebral white matter. Figure 2 illustrated the 12 ROIs that were investigated. Each ROI was sized approximately 30 to 43 mm^2^ and manually and independently placed by Observers 1 and 2 (Authors S.S. and Y.W.) following procedures in classical and standard agreement studies [10]. After an initial consensus decision was drawn regarding the sizes and locations of the 12 ROIs, the observers performed manual segmentations of the ROI independently on each set of images. This ROI placement procedure was repeated by each observer in the following week. 

MTR values were extracted using the manually-defined ROIs with the combinations of observer, time point, and repetition (Table 1). The mean and SDs of the ROI values were calculated. Meta-data were stored in a SAS 9.1 (SAS, Cary, NC, USA) dataset, with individual volunteer identification numbers withheld and replaced by a sequence of 1 to 9 for each subject. 

### 2.4. Statistical Methods

Statistical analyses were performed using SAS 9.1 (SAS Institute, Cary, NC, USA; http://www.sas.com). The SAS analytic procedures conducted included “Proc Univariate,” “Proc Means,” “Proc Corr,” and “Proc Mixed.” Bar diagrams were constructed using Microsoft Excel (http://www.microsoft.com). Age and gender were not controlled for in analyses. 

#### 2.4.1. Descriptive Statistics

Let *Y* = *Y*_*ijklm*_ having the indices described in Table 1 be a random variable representing the mean ROI value. For the *m*th ROI, we first computed the sample mean and standard deviation of all mean ROI values:

(2) $$\begin{array}{cc} \hat{\text{Mean}}\left(Y_{m}\right) & =\bar{Y_{••••m}}=\frac{1}{N_{m}}\overset{2}{\underset{l=1}{\sum }}\overset{2}{\underset{k=1}{\sum }}\overset{2}{\underset{j=1}{\sum }}\overset{9}{\underset{i=1}{\sum }}Y_{ijklm},\\ \hat{\text{SD}}\left(Y_{m}\right) & ={\left\{\hat{\mathrm{Var}\;}(Y_{m})\right\}}^{1/2}\\ & ={\left\{\frac{1}{N_{m}-1}{\overset{2}{\underset{l=1}{\sum }}\overset{2}{\underset{k=1}{\sum }}\overset{2}{\underset{j=1}{\sum }}\overset{9}{\underset{i=1}{\sum }}\left(Y_{ijklm}-\bar{Y_{••••m}}\right)}^{2}\right\}}^{1/2}, \end{array}$$

where *N*_*m*_ = *I* × *J* × *K* × *L* = 9 × 2^3^ = 72 measurements and the operator “•” means the marginal sum over the particular index. 

The 95-percentile normality range was approximately within the following interval, with the following lower and upper bounds:

(3) $$\left(\hat{\text{Mean}}\left(Y_{m}\right)-2\times \hat{\text{SD}}\left(Y_{m}\right),\;\;\hat{\text{Mean}}\left(Y_{m}\right)+2\times \hat{\text{SD}}\left(Y_{m}\right)\right).$$

The term “normality range” as used in Europe, could be arbitrarily-defined according to the number of standard deviations away from the mean [11]. Thus, it should not be viewed as the range of the entire dataset, but rather an interval useful for estimating the population value by one or several standard deviations away from the mean. Here the critical value of 2 was chosen as recommended by Bland and Altman [12]. 

Additionally, we justified using a Student's *t*-distribution with *N*_*m*_ − 1 = 71 degrees of freedom. For any tail probability of *α*/2 (e.g., 0.025 for a 95-percent normality range), we used the quantile of the corresponding to particular *t*-distribution, such that

(4) $$t_{N_{m}-1}^{-1}\left(1-\frac{\alpha }{2}\right)=t_{71}^{-1}\left(0.975\right)=1.994,$$

This value happened to be close to the recommended multiplier of 2. Therefore, we rounded it to 2 in (3) for convenience.

#### 2.4.2. Concordance Using Spearman's Rank Coefficient Coefficients

We first explored and measured the concordance between the various measurements fully nonparametrically via Spearman's rank correlation coefficient. Suppose that we correlated the ROI values by Observers *j* = 1 and *j*′ = 2, then denoted the marginal ranks, *R*_*ijklm*_ = rank_*i*_(*Y*_*ijklm*_) and *R*_*ij*′*klm*_ = rank_*i*′_(*Y*_*i*′*jklm*_), respectively, for all *j* ≠ *j*′ with *j* = 1 and *j*′ = 2. The sample version of Pearson's product-moment correlation coefficient between the ranks of the data was equivalent to Spearman's rank correlation coefficient [13]:

(5) $$\begin{array}{cc} \hat{\text{Cor}}\left(r_{ijklm},r_{ij^{\prime }klm}\right) & =\frac{\left(N_{m}/2\right)\sum_{l=1}^{2}\sum_{k=1}^{2}\sum_{i=1}^{9}\left(R_{i1klm}R_{i2klm}\right)-\sum_{l=1}^{2}\sum_{k=1}^{2}\sum_{i=1}^{9}R_{i1klm}\sum_{l=1}^{2}\sum_{k=1}^{2}\sum_{i=1}^{9}R_{i2klm}}{{\left\{\left(N_{m}/2\right)\sum_{l=1}^{2}\sum_{k=1}^{2}\sum_{i=1}^{9}R_{i1klm}^{2}-𝒜\right\}}^{1/2}{\left\{\left(N_{m}/2\right)\sum_{l=1}^{2}\sum_{k=1}^{2}\sum_{i=1}^{9}R_{i2klm}^{2}-ℬ\right\}}^{1/2}},\\ & =\frac{\sum_{l=1}^{2}\sum_{k=1}^{2}\sum_{i=1}^{9}\left(R_{i1klm}R_{i2klm}\right)-\left(N_{m}/2\right)\bar{R_{i1klm}}\bar{R_{i2klm}}}{\left(N_{m}/2-1\right)\text{SD}\left(R_{i1klm}\right)\text{SD}\left(R_{i2\text{k}lm}\right)}. \end{array}$$

where *𝒜* denotes (∑_*l* = 1_^2^∑_*k* = 1_^2^∑_*i* = 1_^9^ *R*_*i*1*klm*_)^2^ and *ℬ* denotes (∑_*l* = 1_^2^∑_*k* = 1_^2^∑_*i* = 1_^9^ *R*_*i*2*klm*_)^2^. 

Assuming that there was no presence of any ties since the ROI values were of continuous random variables, the Spearman's rank correlation coefficient between Observers *j* and *j*′ was

(6) $$\text{Corr}\left(r_{ijklm},r_{ij^{\prime }klm}\right)=1-\frac{6\sum_{l=1}^{2}\sum_{k=1}^{2}\sum_{i=1}^{9}D_{i•klm}^{2}}{\left(N_{m}/2\right)\left(N_{m}^{2}/4-1\right)},$$

where the difference of an arbitrary pair of marginal ranks for Observer *j* and *j*′ was denoted by *D*_*i*•*klm*_ = *R*_*ijklm*_ − *R*_*ij*′*klm*_, for all *j* ≠ *j*′. Consequently, all of the raw mean ROI values were converted to their marginal ranks and the differences between the ranks of each observation on the two variables were computed. Spearman's rank correlation coefficient was also computed for the ROI values between any two different time points *k* = 1 and *k*′ = 2. 

The strength of the concordance and the benchmark values have been discussed [14]. Bar diagrams were made to display the Spearman's rank correlation coefficients between observers or time points for each ROI.

#### 2.4.3. Reproducibility Using Coefficients of Variations

We used the normalized measure of dispersion of a distribution to evaluate the reproducibility of the measurement [15]. The measure was the coefficient of variation (CV), defined as the ratio of the SD to the mean.

(7) $$\hat{\text{CV}}\left(Y_{m}\right)=\frac{\hat{\text{SD}}\left(Y_{m}\right)}{\hat{\text{Mean}}\left(Y_{m}\right)},$$

where both the numerator (i.e., sample SD) and the denominators (i.e., sample mean) in the above expression for CV are provided in (2). Skewed data, such as those generated by an exponential distribution for which the underlying population mean and standard deviation would be equal, and thus the CV became 1. Hence, CV < 1 would generally represent low variability, and CV > 1 would represent high variability. As in (4) and (6), further stratified computations of CV for different observers, time point, or repetitions were achieved using formulae similar to (7).

#### 2.4.4. Normality and Significance Tests for the Effects via a Multivariate Regression Analysis

As overall variability was likely a result of the effects illustrated in Table 1. We employed a multivariate mixed-effects regression analysis to direct model the ROI values. 

A variance-component approach has advantages over many stratified analyses, especially studying studies with a limited sample size. Here, because of the novel imaging modality using MT and 3T acquisitions with labor-intensive manual segmentation procedures, large number of subjects would not have been feasible. To conduct an analysis of variance (ANOVA) based on the various effects, a distributional assumption of normality was necessary and convenient. Therefore, we conducted marginal normality tests using the Shapiro-Wilk test [16]. We would demonstrate (see Section 3.4) that the normality assumption was generally satisfactory. 

Thus, we could then consider adopting a linear random-effects model with all pair-wise interactions, in addition to a third-order interaction term:

(8) $$\begin{array}{cc} Y_{ijklm} & =\mu_{m}+S_{i}+O_{j}+T_{k}+R_{l}\\ & \;+S_{i}\times O_{j}+S_{i}\times T_{k}+S_{i}\times R_{j}+O_{j}\times T_{k}\\ & \;+O_{j}\times R_{l}+T_{k}\times R_{j}+O_{j}\times T_{k}\times R_{l}+\varepsilon_{ijklm},\\ & \;\;\forall i=1,\ldots ,9,\;j=1,2,\;\;k=1,2,\;\;l=1,2. \end{array}$$

The effects represented the following: *μ*_*m*_ as intercept, *S*_*i*_ as subjects, *O*_*i*_ as observers, *T*_*i*_ as time points, *R*_*i*_ as repetitions, and *ε*_*ijklm*_ as the error team. A random-effects model assumed that each of the effects would have independent normal distributions with mean and variance. 

If normality had failed and because the data were mean ROI values that were positively-valued, we would recommend a Box-Cox transformation, *h*(*Y*_*ijklm*_, *λ*), of the outcome variable with an optimal power coefficient *λ* [17–19]. Note that the log-normal becomes a special case when the power coefficient *λ* = 0. This normality transformation is given by:

(9) $$\begin{array}{cc} Y_{ijklm}^{\prime } & =h\left(Y_{ijklm},\lambda \right)=\{\begin{array}{cc} \frac{{Y_{ijklm}}^{\lambda }-1}{\lambda }, & \lambda \neq 0\\ \log\;\left({Y_{ijklm}}^{\lambda }\right), & \lambda =0 \end{array}\\ & \forall i=1,\ldots ,9,\;\;j=1,2,\;\;k=1,2,\;\;l=1,2. \end{array}$$

A profile log-likelihood, llik of *λ* given the observations *y*_*ijklm*_, would be maximized to estimate an optimal Box-Cox transformation via a nonlinear minimization routine, where the log-likelihood was

(10) $$\begin{array}{cc} & \text{llik}\left(\lambda |y_{ijklm}\right)\\ & =-N_{m}\log\;\left\{SD\left(y_{ijklm}^{\prime }\right)\right\}+\left(\lambda -1\right)\left\{\overset{N_{m}}{\underset{i=1}{\sum }}\log\;\left(y_{ijklm}^{\prime }\right)\right\}+c, \end{array}$$

where *c* was a constant free of the power coefficient to be optimized. 

Due to the limited number of subjects, however, even with an optimal normality transformation, over-fitting and non-convergence might be issues. Alternatively, we could regard all of the observers, time points, and repetitions as fixed and specify a mixed-effects model. The significances of the sources of variability were tested via a restricted maximum likelihood (REML) approach. For our multivariate analysis, the significance threshold for two-tailed *P*-values was set if *P* ≤ .05.

#### 2.4.5. Interobserver Reliability Using the ICCs

Stratified by the time points within each ROI, a two-way ANOVA was performed by regarding all of the observers, time points, and repetitions as fixed. We specified a mixed-effects model for simplicity. Due to the complexity of the variance components, we instead adopted a hybrid approach by considering two effects at once. For example, all subjects were segmented by the same observers who were from an entire population of observers. In other words, the subject effect was always assumed to be random, while the remaining effect (e.g., here the observer) was assumed to be fixed. We computed the Case-3 ICCs, accordingly [20]. 

We simplified our notations by only keeping the indices for the subject and observer effects of interest. We decomposed the data as follows:

(11) $$Y_{ij}=\mu +S_{i}+o_{j}+S_{i}\times o_{j}+\varepsilon_{ij},\;\forall i=1,\ldots ,9,\;\;j=1,2,$$

where the subject effect *S*_*i*_ was assumed to be random in an upper-case letter, which had a normal distribution with mean 0 and variance *σ*_*S*_^2^, for all *i* = 1,…, *I* (here *I* = 9); the observer effect *o*_*j*_ was considered to be a fixed effect in a lower-case letter, with the constraint ∑_*j* = 1_^*J*^ *o*_*j*_ = 0, with the corresponding parameter to the variance being *θ*_*o*_^2^ = (1/(*J* − 1))∑_*j* = 1_^*J*^ *o*_*j*_^2^, for all *j* = 1,…, *J* (here *J* = 2); the interaction term between the subject and the observer *S*_*i*_ × *o*_*j*_ was the degree to which the *j*th observer departed from his or her usual rating tendencies for the *i*th subject, which had a normal distribution with a mean of 0 and variance *σ*_*S*×*o*_^2^; the errors terms *ε*_*ij*_ were assumed to have an independent and identical distribution (iid) normal distribution with a mean of 0 and variance *σ*_*E*_^2^. For the same *i*th subject, the effects are further assumed to be subjected to the constraint ∑_*j* = 1_^*J*^(*S*×*o*)_*ij*_ = 0 over all of the observers. The corresponding two-way ANOVA table was listed (Table 3). 

Shrout and Fleiss gave the true definition of ICC using the variance ratio of the subject variance over the total variance, with its estimated version using the quantities via ANOVA (Table 3) [19]:

(12) $$\begin{array}{c} \text{ICC}=\frac{\sigma_{S}^{2}-\sigma_{S\times o}^{2}/\left(J-1\right)}{\sigma_{S}^{2}+\sigma_{S\times o}^{2}+\sigma_{E}^{2}},\\ \hat{\text{ICC}}\left(3,1\right)=\frac{\text{BSMS}-\text{EMS}}{\text{BSMS}+\left(J-1\right)\text{EMS}}. \end{array}$$

#### 2.4.6. Intraobserver Reliability Using the ICCs

Similar to the analysis described above, we adopted a hybrid approach by considering two effects at once, with the subject effect always assumed to be random and the time point assumed to be fixed. The associate model was given by

(13) $$Y_{ij}=\mu +S_{i}+t_{k}+S_{i}\times t_{k}+\varepsilon_{ik},\;\forall i=1,\ldots ,9;\;\;k=1,2.$$

As in (12), the estimated intraobserver agreement and its estimate were provided by:

(14) $$\begin{array}{c} \text{ICC}=\frac{\sigma_{S}^{2}-\sigma_{S\times t}^{2}/\left(K-1\right)}{\sigma_{S}^{2}+\sigma_{S\times t}^{2}+\sigma_{E}^{2}},\\ \hat{\text{ICC}}\left(3,1\right)=\frac{\text{BSMS}-\text{EMS}}{\text{BSMS}+\left(K-1\right)\text{EMS}}, \end{array}$$

where the interaction term the interaction term between the subject and the time *S*_*i*_ × *t*_*k*_ had a normal distribution with a mean of 0 and variance *σ*_*S*×*t*_^2^.

#### 2.4.7. Sensitivity Analyses of the ICCs under Various Models

We performed a sensitivity analysis by computing 6 different ICC values Shrout and Fleiss previously proposed assumptions for ICCs (Table 4) [18]. A SAS macro, written by Professor Robert Hamer, University of North Carolina School of Medicine, Chapel Hill, NC, USA (http://www.bios.unc.edu/~hamer), was run to perform the various ICC computations.

## 3. Results

### 3.1. Descriptive Statistics

Eleven healthy adults provided written informed consent to be evaluated and 9 underwent brain scans. Mean age of participants who received scans was 37.9 ± 14.2 years; 7 participants were men and 2 were women. 

The mean ROI values varied across different region (Table 5). The left and right hemispheres tended to yield similar results when the average over these healthy subjects was considered.

### 3.2. Concordance Using Spearman's Rank Coefficient Coefficients

**Figure 3:**
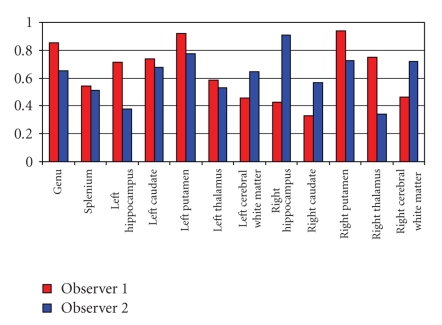
Spearman's rank correlation coefficients between the two different time points for the same observer (red = Observer 1; blue = Observer 2).

**Figure 4:**
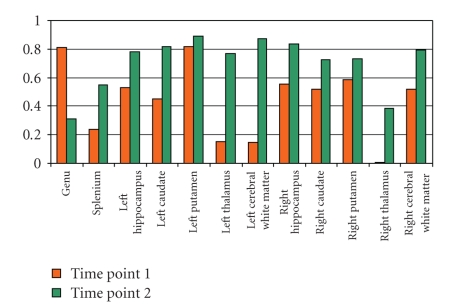
Spearman's rank correlation coefficients between the two different observers for the same time point (orange = Time Point 1; green = Time Point 2).

Spearman's rank correlation coefficients showed that a majority of correlations within each observer was above 0.5, suggesting a moderate to high concordance (Figure 3). Time point 2 tended to yield higher concordance between the observers, which suggested a possible learning effect over time (Figure 4). Due to limited sample sizes in this pilot study, in Figures 3 and 4, we demonstrated the effect of observers by averaging over repetitions by each observer. Similarly, we demonstrated the effect of time points by averaging over repetitions at each time point.

### 3.3. Reproducibility Using Coefficients of Variations

Overall, CVs ranged from 1.2% in the genu for Observer 2 to 7.0% in the right hippocampus for Observer 1 (Table 6). Since all of the CVs were within 7%, that is, all CVs were less than 10%, the reproducibility was reasonably high.

### 3.4. Normality and Significance Tests via a Multivariate Analysis

The tests of the normal distribution assumption marginally using the Shapiro-Wilk test indicated that only occasionally (e.g., for left caudate, left and right putamen, and right hippocampus), this assumption was not met (see Table 7). Therefore, it was reasonable to specify linear mixed-effects modeling and two-way ANOVA reported in Sections 3.5 and 3.6. 

### 3.5. Interobserver Reliability Using the ICCs

At time point 1, ICCs were greater than 0.7 in regions of genu, left and right putamen, whereas ICCs were from 0.5 to 0.7 in regions of splenium, left and right hippocampus, left caudate, and right cerebral white matter (Table 8). These results indicated moderate to strong interobserver reliability. In comparison, at time point 2, ICCs were greater than 0.7 in regions of genu, splenium, left and right caudate, putamen and cerebral white matter, and left hippocampus and thalamus, while ICCs were from 0.5 to 0.7 in right hippocampus and thalamus. These results suggested a learning effect over time. However, for some ROIs such as the left cerebral white matter, right caudate, right thalamus, ICCs increased from 0.2 (at time point 1) to 0.9 (at time point 2), making it difficult to determine whether this represents a learning effect. 

### 3.6. Intraobserver Reliability Using the ICCs

At each time point, intraobserver agreement was at least 0.5 for a majority of the regions (Table 9). 

### 3.7. Sensitivity Analyses of the ICCs under Various Models

Six different methods for generating ICCs exhibited similar patterns for high vs. low reliability results in different ROIs (Table 10). Thus, reliability appeared to be sensitive to ROI.

## 4. Conclusions and Discussion

We present mathematical methods for MT brain images using 3-T high resolution. Our image analysis may provide useful pilot information for future investigations. These mathematical and statistical methods may easily be generalized to practical studies with larger sample sizes or to studies of patients with active disease. 

We acquired repeat brain measurements based on a high resolution MT imaging protocol at 3T in 9 healthy adults. Our results indicate moderate to high reproducibility, supporting the validity of this method for further studies. Overall, higher intraobserver reliability was observed at the second time point than that at the initial time point, suggesting a possible learning curve effect for both observers. Interobserver reliability was generally lower than intraobserver variability, suggesting a strong observer effect in this comparison, which may be a factor in future investigations using MT imaging. 

Our analyses examined different aspects in a typical observer-agreement study, using measures for concordance, reproducibility, reliability, variance-component analysis, and multivariate analysis. In other studies, all or some of such methods may be considered. However, with a simpler study of either several observers, or one observer with several repetitions at different sessions or time points, then these scenarios may only require several of our methods. Only a small sample of healthy volunteers was evaluated in this initial pilot study. Therefore, the generalization of the 95-percentile normality range may be limited with respect to the wider spectrum of brain mechanisms represented in the broader population. For instance, demonstrating summary measures using all possible observer and time point combinations may not lead to meaningful interpretations in all cases. Nevertheless, since the technology is new, this research may provide useful pilot information for future investigations. Moreover, the statistical methods employed and illustrated here may easily be generalized to studies with larger sample sizes and diseased subjects. 

Another limitation was that this study aimed to evaluate only the reproducibility and reliability, rather than the accuracy in a more comprehensive validation study. In the absence of a true gold standard, such as one based on digital phantoms where realistic variability may still not be simulated, or on histopathology, improved reliability may not be equated with improved accuracy [21]. Both sensitivity and specificity are of interest. Further research would benefit from a useful algorithm to perhaps statistically and optimally estimate the underlying spatial “ground truth” [22, 23]. 

Finally, future research may be directed to evaluating the diagnostic utility of high resolution MT for early detection of Alzheimer's disease, multiple sclerosis or other neurological disorders and for monitoring progression across the clinical course. 

## Figures and Tables

**Table 1:** The random or fixed effects in the data structure for the repeated measures MT study.

Outcome Variable *Y*_*ijkln*_	Effect in the Variance-Component Analysis	Type of Effect	Mathematical Symbol	Index	Maximum of the Index
Mean ROI Value via Manual Segmentations	Subject	Random	*S*_*i*_	*i* = *i*,…, *I*	*I* = 9
	Observer	Fixed or Random	*O*_*j*_	*j* = 1,…, *J*	*J* = 2
	Time Point	Fixed or Random	*T*_*k*_	*k* = 1,…, *K*	*K* = 2
	Repetition	Fixed or Random	*R*_*l*_	*l* = 1,…, *L*	*L* = 2
	Region of Interest	Fixed	*K*_*m*_	*m* = 1,…, *M*	*M* = 12
	Interaction Terms	Generally Mixed	{*S*_*i*_; *O*_*j*_; *T*_*k*_; *R*_*l*_; *K*_*m*_}	{*i*; *j*; *k*; *l*; *n*}	Based on the Appropriate Model Specification

**Table 2:** Various strengths of correlation coefficients as a measure of concordance.

Absolute Value of the Correlation Coefficient	Strength of the Concordance Between Samples
0.0	No
0.2	Weak
0.5	Moderate
0.8	Strong
1.0	Perfect

**Table 3:** Two-way ANOVA table for the mixed-effects model.

Source of Variation	Degrees of Freedom	Mean Squares	
(A) Between Subjects	*I* − 1	BSMS	*J* *σ*_*S*_^2^ + *σ*_*E*_^2^
(B) Within Subjects	*I*(*J* − 1)	WSMS	*θ*_*o*_^2^ + *J* *σ*_*S*×*o*_^2^/(*J* − 1) + *σ*_*E*_^2^
(B.1) Between Observers	*J* − 1	OMS	*I* *θ*_*o*_^2^ + *J* *σ*_*S*×*o*_^2^/(*J* − 1) + *σ*_*E*_^2^
(B.2) Error	(*I* − 1)(*J* − 1)	EMS	*J* *σ*_*S*×*o*_^2^/(*J* − 1) + *σ*_*E*_^2^

Note: BSMS: Between Subjects Mean Squares; WSMS: Within Subject Mean Squares; OMS: Observer Mean Squares; EMS: Error Mean Squares.

**Table 4:** Six different ICCs computed via a sensitivity analysis of the modeling choices.

Notation for the ICC Measure	Multivariate Modeling Assumptions
ICC(1,1)	Each subject is rated by multiple observers; the observers are assumed to be randomly assigned to the subjects; all subjects have the same number of observers.
ICC(2,1)	All subjects are rated by the same observers who are assumed to be a random subset of all possible observers.
ICC(3,1)	All subjects are rated by the same observers who are assumed to be the entire population of observers.
ICC(1,2)	Same assumptions as ICC(1,1) but reliability for the mean of 2 ratings.
ICC(2,2)	Same assumptions as ICC(2,1) but reliability for the mean of 2 ratings.
ICC(3,2)	Same assumptions as ICC(3,1) but reliability for the mean of 2 ratings. Assumes additionally there is no subject × observer interaction.

**Table 5:** Descriptive statistics and 95-percentile normality range of mean ROI values.

Region of Interest	Descriptive Statistics (Mean ± SD)	95% Normality Range (Mean ± 2 × SD)
Genu	77.0 ± 1.0	75.0–79.0
Splenium	72.8 ± 1.5	69.9–75.7
Left Hippocampus	51.5 ± 2.5	46.6–56.4
Left Caudate	59.5 ± 2.2	55.2–63.8
Left Putamen	62.0 ± 2.0	58.1–65.9
Left Thalamus	61.6 ± 2.3	57.1–66.1
Left Cerebral White Matter	73.2 ± 1.2	70.8–75.6
Right Hippocampus	52.0 ± 3.3	45.5–58.5
Right Caudate	61.3 ± 1.7	58.0–64.6
Right Putamen	62.8 ± 1.5	59.9–65.7
Right Thalamus	61.1 ± 2.5	56.2–66.0
Right Cerebral White Matter	73.0 ± 1.3	70.5–75.5

Note: Results were pooled among all 72 observations within each region of interest. SD: standard deviation.

**Table 6:** Coefficient of Variation (CV) of the mean Region of Interest values for each observer.

Region of Interest	Observer 1		Observer 2	
	Mean ± SD (*N* = 36)	CV (%)	Mean ± SD (*N* = 36)	CV (%)
Genu	76.9 ± 1.0	1.3	77.1 ± 0.9	1.2
Splenium	73.1 ± 1.4	1.9	72.6 ± 1.5	2.1
Left Hippocampus	51.3 ± 2.4	4.7	51.6 ± 2.7	5.2
Left Caudate	59.7 ± 1.9	3.2	59.3 ± 2.5	4.2
Left Putamen	61.9 ± 2.2	3.6	62.1 ± 1.9	3.1
Left Thalamus	59.9 ± 1.5	2.5	63.3 ± 1.7	2.7
Left Cerebral White Matter	73.3 ± 1.3	1.8	73.1 ± 1.2	1.6
Right Hippocampus	52.5 ± 3.7	7.0	51.5 ± 2.7	5.2
Right Caudate	61.2 ± 1.9	3.1	61.5 ± 1.4	2.3
Right Putamen	62.7 ± 1.5	2.4	62.8 ± 1.5	2.4
Right Thalamus	59.7 ± 1.7	2.8	62.5 ± 2.5	4.0
Right Cerebral White Matter	73.2 ± 1.2	1.6	72.8 ± 1.4	1.9

Note. SD: standard deviation.

**Table 7:** *P*-value from the Shapiro-Wilk test of marginal normal distributions.

Region of Interest	*P*-value		*P*-value	
	Time Point 1		Time Point 2	
	Observer 1	Observer 2	Observer 1	Observer 2
Genu	.29	.17	.70	.36
Splenium	.31	.06	.93	.61
Left Hippocampus	.14	.81	.45	>.99
Left Caudate	.97	<.0001^a^	.49	.92
Left Putamen	.20	.06	.01^a^	.01^a^
Left Thalamus	.86	.51	.63	.13
Left Cerebral White Matter	.82	.43	.21	.02
Right Hippocampus	.54	.86	.01^a^	.58
Right Caudate	.49	.80	.60	.89
Right Putamen	.07	.003^a^	.25	.03^a^
Right Thalamus	.50	.68	.82	.13
Right Cerebral White Matter	.79	.78	.16	.54

^a^Normal distribution was not met.

**Table 8:** Interobserver reliability between two observers for each time point.

Region of Interest	Inter-Reader ICC	Inter-Reader ICC
	Time Point 1	Time Point 2
Genu	0.866	0.726
Splenium	0.537	0.758
Left Hippocampus	0.693	0.796
Left Caudate	0.580	0.902
Left Putamen	0.869	0.962
Left Thalamus	0.410	0.855
Left Cerebral White Matter	0.378	0.929
Right Hippocampus	0.653	0.656
Right Caudate	0.209	0.872
Right Putamen	0.725	0.882
Right Thalamus	0.264	0.572
Right Cerebral White Matter	0.637	0.896

**Table 9:** Intraobserver reliability within each observer between different repetitions.

Region of Interest	Intraobserver ICC	Intraobserver ICC
	Observer 1	Observer 2
Genu	0.537	0.555
Splenium	0.598	0.756
Left Hippocampus	0.520	0.596
Left Caudate	0.709	0.362
Left Putamen	0.940	0.784
Left Thalamus	0.479	0.622
Left Cerebral White Matter	0.560	0.703
Right Hippocampus	0.411	0.826
Right Caudate	0.473	0.436
Right Putamen	0.659	0.657
Right Thalamus	0.687	0.308
Right Cerebral White Matter	0.570	0.770

**Table 10:** Sensitivity analysis of 6 different interobserver ICCs.

Region of Interest	ICC (1,1)	ICC (2,1)	ICC (3, 1)	ICC (1, 2)	ICC (2, 2)	ICC (3, 2)
Interobserver ICC at Time 1						

Genu	0.870	0.879	0.866	0.931	0.935	0.928
Splenium	0.497	0.463	0.537	0.664	0.633	0.699
Left Hippocampus	0.653	0.605	0.693	0.790	0.754	0.819
Left Caudate	0.562	0.542	0.580	0.719	0.703	0.734
Left Putamen	0.871	0.874	0.869	0.931	0.933	0.930
Left Thalamus	−0.015	0.114	0.410	−0.030	0.205	0.581
Left Cerebral White Matter	0.382	0.385	0.378	0.553	0.556	0.549
Right Hippocampus	0.660	0.669	0.653	0.795	0.802	0.790
Right Caudate	0.178	0.180	0.209	0.302	0.306	0.346
Right Putamen	0.725	0.732	0.720	0.840	0.845	0.837
Right Thalamus	−0.092	0.079	0.264	−0.202	0.146	0.417
Right Cerebral White Matter	0.630	0.621	0.637	0.773	0.766	0.779

Interobserver ICC at Time 2						

Genu	0.722	0.715	0.726	0.838	0.834	0.841
Splenium	0.758	0.757	0.758	0.862	0.862	0.863
Left Hippocampus	0.792	0.785	0.796	0.884	0.880	0.886
Left Caudate	0.905	0.909	0.902	0.950	0.952	0.949
Left Putamen	0.961	0.959	0.962	0.980	0.979	0.980
Left Thalamus	0.297	0.239	0.855	0.458	0.385	0.922
Left Cerebral White Matter	0.928	0.926	0.929	0.963	0.962	0.963
Right Hippocampus	0.640	0.620	0.656	0.781	0.765	0.793
Right Caudate	0.876	0.884	0.872	0.934	0.938	0.932
Right Putamen	0.884	0.887	0.882	0.938	0.940	0.937
Right Thalamus	0.419	0.347	0.572	0.591	0.516	0.728
Right Cerebral White Matter	0.889	0.876	0.896	0.941	0.934	0.945

**Table 11:** Sensitivity analysis of 6 different intraobserver ICCs.

Region of Interest	ICC (1,1)	ICC (2,1)	ICC (3, 1)	ICC (1, *k*)	ICC (2, *k*)	ICC (3, *k*)
Intraobserver for Observer 1						

Genu	0.537	0.537	0.537	0.699	0.699	0.699
Splenium	0.590	0.579	0.598	0.742	0.733	0.749
Left Hippocampus	0.531	0.544	0.520	0.694	0.705	0.684
Left Caudate	0.704	0.696	0.709	0.826	0.821	0.830
Left Putamen	0.942	0.946	0.940	0.970	0.972	0.969
Left Thalamus	0.481	0.484	0.479	0.650	0.653	0.647
Left Cerebral White Matter	0.550	0.539	0.560	0.710	0.701	0.718
Right Hippocampus	0.426	0.439	0.411	0.597	0.610	0.582
Right Caudate	0.470	0.467	0.473	0.640	0.637	0.643
Right Putamen	0.657	0.654	0.659	0.793	0.791	0.795
Right Thalamus	0.696	0.711	0.687	0.821	0.831	0.814
Right Cerebral White Matter	0.582	0.596	0.570	0.736	0.747	0.727

Intraobserver ICC for Observer 2						

Genu	0.563	0.572	0.555	0.720	0.728	0.714
Splenium	0.760	0.767	0.756	0.864	0.868	0.861
Left Hippocampus	0.607	0.623	0.596	0.756	0.767	0.747
Left Caudate	0.365	0.367	0.362	0.535	0.537	0.531
Left Putamen	0.790	0.800	0.784	0.883	0.889	0.879
Left Thalamus	0.632	0.645	0.622	0.774	0.784	0.767
Left Cerebral White Matter	0.712	0.726	0.703	0.832	0.841	0.826
Right Hippocampus	0.829	0.835	0.826	0.907	0.910	0.905
Right Caudate	0.432	0.429	0.436	0.603	0.601	0.607
Right Putamen	0.667	0.682	0.657	0.800	0.811	0.793
Right Thalamus	0.298	0.294	0.308	0.459	0.455	0.471
Right Cerebral White Matter	0.777	0.789	0.770	0.875	0.882	0.870
